# Searching PubMed during a Pandemic

**DOI:** 10.1371/journal.pone.0010039

**Published:** 2010-04-07

**Authors:** Ole Norgaard, Jeffrey V. Lazarus

**Affiliations:** 1 National Centre for Health Promotion and Disease Prevention, National Board of Health, Copenhagen, Denmark; 2 World Health Organization Regional Office for Europe, Copenhagen, Denmark; 3 Copenhagen School of Global Health, Copenhagen University, Copenhagen, Denmark; Copenhagen University Hospital, Denmark

## Abstract

**Background:**

The 2009 influenza A(H1N1) pandemic has generated thousands of articles and news items. However, finding relevant scientific articles in such rapidly developing health crises is a major challenge which, in turn, can affect decision-makers' ability to utilise up-to-date findings and ultimately shape public health interventions. This study set out to show the impact that the inconsistent naming of the pandemic can have on retrieving relevant scientific articles in PubMed/MEDLINE.

**Methodology:**

We first formulated a PubMed search algorithm covering different names of the influenza pandemic and simulated the results that it would have retrieved from weekly searches for relevant new records during the first 10 weeks of the pandemic. To assess the impact of failing to include every term in this search, we then conducted the same searches but omitted in turn “h1n1,” “swine,” “influenza” and “flu” from the search string, and compared the results to those for the full string.

**Principal Findings:**

On average, our core search string identified 44.3 potentially relevant new records at the end of each week. Of these, we determined that an average of 27.8 records were relevant. When we excluded one term from the string, the percentage of records missed out of the total number of relevant records averaged 18.7% for omitting “h1n1,” 13.6% for “swine,” 17.5% for “influenza,” and 20.6% for “flu.”

**Conclusions:**

Due to inconsistent naming, while searching for scientific material about rapidly evolving situations such as the influenza A(H1N1) pandemic, there is a risk that one will miss relevant articles. To address this problem, the international scientific community should agree on nomenclature and the specific name to be used earlier, and the National Library of Medicine in the US could index potentially relevant materials faster and allow publishers to add alert tags to such materials.

## Introduction

The 2009 pandemic of influenza A(H1N1), first known as swine flu, was initially detected in humans in Mexico in April. Within weeks it had reached the United States and then Europe. As of 10 January 2010, more than 208 countries and overseas territories or communities have reported laboratory confirmed cases. While most cases have been mild, there have been at least 13,500 deaths [1].

In addition to being featured in tens of thousands of newspaper and magazine articles, the influenza A(H1N1) pandemic has been well covered in the scientific literature. For the scientific community to react swiftly and effectively to such a pandemic, it is crucial that all the relevant research and communications published in scientific journals reach experts as quickly as possible.

Colleagues, peers and professional networks are of great importance in meeting the experts' information needs [2]. However, relying solely on these sources will increase the risk of missing important information. To supplement this knowledge transfer, a systematic search of bibliographic databases such as MEDLINE is necessary.

MEDLINE covers over 16 million records of articles published in more than 5,000 international journals in the fields of biomedicine and health. As a complementary interface for searching MEDLINE, PubMed (http://www.pubmed.gov) is a likely first choice for people seeking to monitor medical research on a given topic. PubMed also includes a database of additional material, much of it newly published. Articles that have not yet been added to the MEDLINE database, where everything is indexed with medical subject headings (MeSH), appear in PubMed as soon as publishers provide citation data for published tables of contents, including titles, authors, and in most cases abstracts [3], [4].

MeSH are a controlled vocabulary developed and used by the United States National Library of Medicine (NLM). They consist of sets of descriptors arranged in a hierarchical structure that permits searching at various levels of specificity [5]. Skilled NLM subject analysts examine journal articles and assign them the most specific MeSH applicable – typically 10–12 per record. Applying the MeSH vocabulary ensures that articles are uniformly indexed by subject, whatever the author's keywords [6]. Unfortunately, it can take several months for analysts to assign MeSH.

Articles on the influenza A(H1N1) pandemic are likely to be assigned the MeSH “Influenza A Virus, H1N1 Subtype” as well as “Disease outbreaks.” Combining these two MeSH is expected to generate a highly precise search result. However, if MeSH have not yet been assigned to the articles of interest, PubMed queries that only use MeSH will *not* detect them.

In a pandemic situation, where rapid retrieval of newly published material is essential, identifying articles that have not yet been assigned MeSH poses a significant challenge. It can only be done by searching for the terms used in the citation data provided by the publisher and added to the PubMed database. It is critical to choose terms that maximise the retrieval of records that may be relevant for the work of health experts and policy-makers.

Since the first outbreak in April 2009, a variety of names have been associated with this virus, e.g. swine flu, swine-origin influenza virus (S-OIV), Mexico flu, novel influenza virus, influenza A/H1N1, influenza A(H1N1), H1N1 2009, H1N1/09 and, most recently, pandemic (H1N1) 2009, the term adopted by the World Health Organization (WHO). This variety characterises articles published in scientific journals as well as the popular press.

In this study, we demonstrate the pitfalls of inconsistent naming and the effects it can have for health experts on obtaining relevant scientific information, and we will suggest several strategies to help address the problem.

## Methods

In order to assess the impact of failing to include relevant terms when conducting a search in PubMed, we first developed a search algorithm to simulate searches carried out every week during the first 10 weeks of the pandemic. Such a simulation was necessary to exclude records that would be returned by a search today due to subsequent MeSH indexing, but would not be returned by searches when the pandemic was first unfolding. The searches used here were carried out at the end of August 2009.

### Constructing a core search string

Based on our own monitoring of scientific articles and news media, as well as consultations with influenza experts, primarily from WHO, we identified five terms as relevant and likely to be present in the records of articles on the pandemic: “h1n1,” “swine,” “pandemic,” “epidemic,” and “outbreak.” We considered the last four relevant only if the term “influenza” or “flu” was also present in the database's record of the article. The following steps accordingly made up our core PubMed search string:

#A h1n1
#B swine OR pandemic OR epidemic OR outbreak
#C influenza OR flu
#D #A OR (#B AND #C)

Because of PubMed's automatic term-mapping feature, certain terms are “translated” when searches are carried out [6]. For the above search, the following translations apply. (See Box S1 for an explanation of the search tags we used.)

- *h1n1*
  “h1n1”[all]
- *swine*
  “Swine”[mh] OR “swine”[all] OR “sus scrofa”[mh] OR (“sus”[all] AND “scrofa”[all]) OR “sus scrofa”[all]
- *pandemic*
  “Disease Outbreaks”[mh] OR (“disease”[all] AND “outbreaks”[all]) OR “disease outbreaks”[all] OR “pandemic”[all]
- *epidemic*
  “Disease Outbreaks”[mh] OR (“disease”[all] AND “outbreaks”[all]) OR “disease outbreaks”[all] OR “epidemic”[all]
- *outbreak*
  “Disease Outbreaks”[mh] OR (“disease”[all] AND “outbreaks”[all]) OR “disease outbreaks”[all] OR “outbreak”[all]
- *influenza*
  “Influenza, Human”[mh] OR (“influenza”[all] AND “human”[all]) OR “human influenza”[all] OR “influenza”[all]
- *flu*
  “Influenza, Human”[mh] OR (“influenza”[all] AND “human”[all]) OR “human influenza”[all] OR “flu”[all]

This translation ensures that records indexed under the included MeSH “Disease Outbreaks,” “Influenza, Human” or “Swine” are identified even if they do not contain any of the actual terms used in the search algorithm. It is worth noting that the MeSH “Influenza, Human,” which both “influenza” and “flu” translate into, does not include the MeSH “Influenza A Virus, H1N1 Subtype” as these MeSH are placed in two separate branches of the vocabulary's hierarchical structure. However, including the term “h1n1”[all] or “influenza”[all] will return records assigned the latter MeSH because it contains both “h1n1” and “influenza.”

### Simulating a historical search

Using the core search string, we built additional strings to simulate a search carried out at the end of a given week for relevant materials that had been added to PubMed during that week. We used weeks that ran from Monday to Sunday, beginning with the last week of April 2009, meaning that Week 1 was 27 April–3 May 2009, Week 2 was 4–10 May 2009, and so on.

To find all the records available at the end of Week 1, we first used PubMed to identify all records returned by the core search string:

#1 h1n1 OR ((swine OR pandemic OR epidemic OR outbreak) AND (influenza OR flu))

We then limited the records to the ones entered into PubMed during Week 1:

#2 2009/04/27:2009/05/03[edat]
#3 #1 AND #2

For the next part of the algorithm, we looked for records with a MeSH date (see Box S1) between 27 April and 3 May 2009, which identified all records that had been assigned MeSH during Week 1 or had been entered into PubMed in Week 1 but not yet assigned MeSH at the time of our simulated search.

#4 2009/04/27:2009/05/03[mhda]
#5 #3 AND #4

Then we isolated the records that have been assigned MeSH *after* Week 1, i.e. later than 3 May 2009:

#6 2009/05/04:2099/12/31[mhda]
#7 #3 AND #6

To these we applied the core search string as if no MeSH had been assigned to the records, to reflect the delay in assigning such headings. In a typical search conducted at the time, almost all the database fields would have been searched, including MeSH (see the description of the search tag [all] in Box S1). Ideally, we would restrict the search to all database fields besides MeSH, but PubMed does not provide a possibility to exclude a search tag, such as [mh]. Instead, we limited our search to the fields “Title” and “Abstract” (by using the search tag [tiab]), assuming that no other fields would contain any information that would retrieve a record if a title and abstract search did not already identify it.

As the automatic translation produces a string that includes additional terms, we included them explicitly in our string. However, we removed terms that would produce results identical to other terms in the string, such as “sus scrofa,” “disease outbreaks,” and “human influenza.” The resulting search simulated one in which MeSH have not yet been assigned to the records.

#8 h1n1[tiab] OR ((swine[tiab] OR (sus[tiab] AND scrofa[tiab]) OR pandemic[tiab] OR epidemic[tiab] OR outbreak[tiab] OR (disease[tiab] AND outbreaks[tiab])) AND (influenza[tiab] OR flu[tiab] OR (influenza[tiab] AND human[tiab])))
#9 #7 AND #8

Finally, we joined the results of our search for records that have not yet been assigned MeSH or where this happened during Week 1 with the records assigned MeSH after Week 1:

#10 #5 OR #9

The end result simulates a search carried out at the end of the last day of Week 1, i.e. 3 May 2009, for potentially relevant publications added to the PubMed database during the previous week.

### Identifying relevant records

By reviewing the titles and available abstracts of all the potentially relevant records returned by Step 10, we identified the ones relevant to the 2009 influenza A(H1N1) pandemic. We conducted this review individually, and then the two of us conferred on which records to classify as relevant.

We included as relevant articles accepted for publication or published before the first outbreak was known if they were on subjects such as influenza vaccine development, oseltamivir (Tamiflu) resistance, or general influenza pandemic preparedness. We excluded articles primarily on influenza A(H5N1) (avian influenza) if they contained no obvious linkages to one of these subjects.

We considered all types of publications for classification as relevant, including publications categorised by the NLM as “news,” which covers announcements, statements of new data, reports of recent events, and other matters of interest to the field of science. *Nature* and *Science* are two examples of journals publishing substantive news reports of vitally important and sometimes controversial developments, often with data [7].

Then we incorporated the ID of every relevant record in a single search string. This query produced a list of the relevant records that would have been found if the core search string (#1) was used in a PubMed search at the end of a given week, e.g. at the close of 3 May 2009, for articles added to PubMed during that week.

#11 19408352[pmid] OR 19407756[pmid] OR 19407739[pmid]…

### Identifying missed relevant records

To show how many relevant records would not have been identified if one of the key terms – “h1n1,” “swine,” “influenza,” or “flu” – were left out of the search string, we carried out a search that was similar to the above but excluded each term in turn, and then we compared the results. The following example excludes “h1n1”:

#12 (swine OR pandemic OR epidemic OR outbreak) AND (influenza OR flu)
#13 (swine[tiab] OR (sus[tiab] AND scrofa[tiab]) OR pandemic[tiab] OR epidemic[tiab] OR outbreak[tiab] OR (disease[tiab] AND outbreaks[tiab])) AND (influenza[tiab] OR flu[tiab] OR (influenza[tiab] AND human[tiab]))
#14 (#2 AND #4 AND #12) OR (#2 AND #4 AND #13)
#15 (#10 NOT #14) AND #11

We carried out similar searches that excluded the other key terms in turn. For “swine,” the term itself was excluded as well as traces produced by the automatic translation, (“swine”[tiab] OR (“sus”[tiab] AND “scrofa”[tiab])). For “influenza” and for “flu,” we only excluded the term itself, since in both cases the translation adds (“influenza”[tiab] AND “human”[tiab]) to the search string.

As a tool for additional analysis of parts of the results we extracted the publication types assigned to the records. We divided the publication types relevant for this study into two categories:

- **Publication category A**: case report, clinical trial, comparative study, evaluation study, journal article, multicenter study, research support and review;
- **Publication category B**: comment, congress report, editorial, historical article, interview, letter, news and newspaper article.

Records can be assigned one or more publication types. This is not necessarily done at the same time as MeSH are added.

## Results

To calculate the proportion of relevant records missed by each partial search, we first tallied the number of records identified at two different steps of the search process (see Table 1, Columns A and B). The percentages of relevant records that would have been missed if a given term were not included are shown in Table 1, Column C.

**Table 1:** Search results by week (27 April–5 July 2009).

	A. Potentially relevant records	B. Relevant records	C. Relevant records missed when the given term was omitted from the search			
			“h1n1”	“swine”	“influenza”	“flu”
Week 1	40	25	0.0% (0)	20.0% (5)	28.0% (7)	36.0% (9)
Week 2	29	20	20.0% (4)	10.0% (2)	5.0% (1)	30.0% (6)
Week 3	48	29	27.6% (8)	6.9% (2)	20.7% (6)	6.9% (2)
Week 4	49	27	7.4% (2)	3.7% (1)	37.0% (10)	22.2% (6)
Week 5	43	27	22.2% (6)	22.2% (6)	22.2% (6)	25.9% (7)
Week 6	43	25	24.0% (6)	16.0% (4)	12.0% (3)	16.0% (4)
Week 7	35	24	16.7% (4)	16.7% (4)	8.3% (2)	16.7% (4)
Week 8	36	21	23.8% (5)	23.8% (5)	4.8% (1)	23.8% (5)
Week 9	84	57	36.8% (21)	3.5% (2)	10.5% (6)	7.0% (4)
Week 10	36	23	8.7% (2)	13.0% (3)	26.1% (6)	21.7% (5)
**Total**	**443**	**278**	**58**	**34**	**48**	**52**
**Mean**	**44.3**	**27.8**	**18.7% (5.8)**	**13.6% (3.4)**	**17.5% (4.8)**	**20.6% (5.2)**

A: Number of potentially relevant new records in PubMed at the end of each week, as identified by a simulated historical search (Step 10).

B: Number of records identified as relevant after review of all records in A (Step 11).

C: Percentage of relevant records in Column B missed when a given search term was not included, with the number of records in parentheses (Step 15). The mean percentages and records are calculated as an arithmetic mean of the weekly values.

Using our core search string at the end of each week, we identified an average of 44.3 potentially relevant records each week (range 29–84 records). Out of these, we found an average of 27.8 records (range 20–57 records) to be relevant.

When we excluded one of the terms from the search string, the average percentage of relevant records that we missed was 18.7% (range 0.0%–36.8%) for “h1n1,” 13.6% (range 3.5%–23.8%) for “swine,” 17.5% (range 4.8%–37.0%) for influenza, and 20.6% (range 6.9%–36.0%) for “flu.”

**Figure 1:**
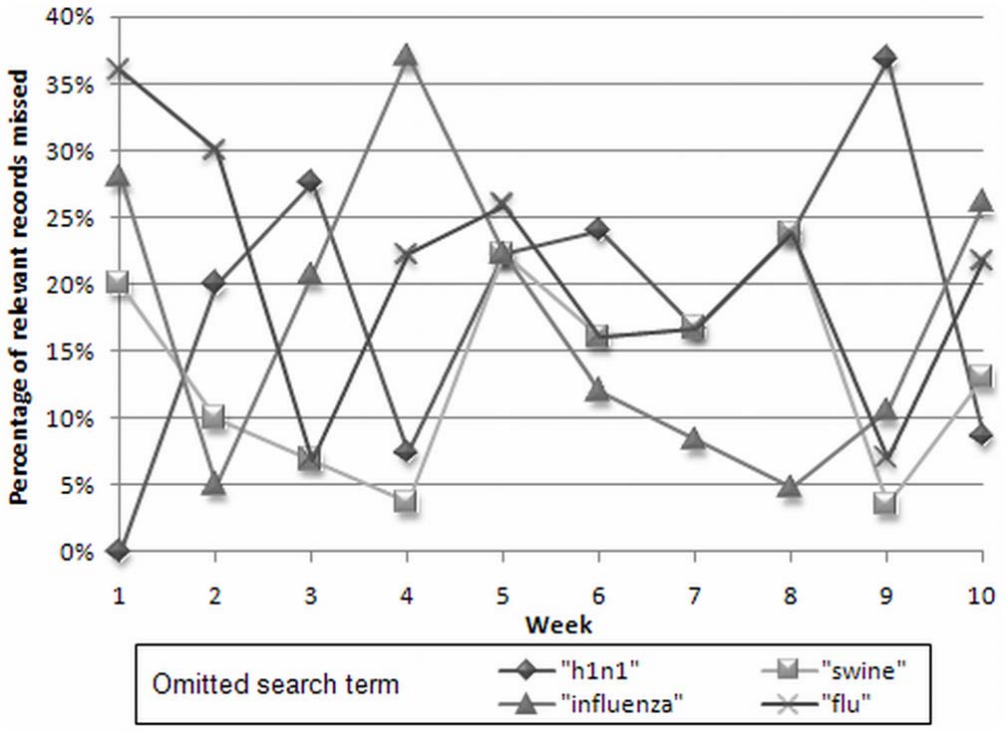
Percentage of relevant records missed when a key search term is omitted, Weeks 1–10.

Figure 1 shows that the consequences of omitting a key search term vary considerably, with identification failure rates ranging from 0.0% (for leaving out “h1n1” in Week 1) to 37.0% (for “influenza” in Week 4).

Table 2 lists a few examples of the relevant records that were missed in searches carried out at different points in time.

**Table 2:** Examples of missed relevant records.

Term excluded from search	Title of record missed by search (journal title)	Publication type
“h1n1”	**H1N1 influenza A disease – information for health professionals** (The New England Journal of Medicine) [15]	Editorial
	**Serum cross-reactive antibody response to a novel influenza A (H1N1) virus after vaccination with seasonal influenza vaccine** (Morbidity and Mortality Weekly Report) [16]	Journal article
“swine”	**Obstetrical concern on new emerging swine flu** (Archives of Gynecology and Obstetrics) [17]	Journal article
	**Swine flu: some good lessons learnt** (British Journal of Community Nursing) [18]	Journal article
“influenza”	**Reported changes in health-related behaviours in Chinese urban residents in response to an influenza pandemic** (Epidemiology and Infection) [19]	Journal article
	**Swine-origin influenza virus in young age groups** (The Lancet) [20]	Letter
“flu”	**Prisons' preparedness for pandemic flu and the ethical issues** (Public Health) [21]	Journal article
	**Public perceptions, anxiety, and behaviour change in relation to the swine flu outbreak: cross sectional telephone survey** (BMJ) [22]	Journal articleMulticenter studyResearch support

Table 3 shows the number of articles (all types) missed by journal as well as the number missed that were classified by us as publication category A. In 11 of the 16 journals that had published three or more relevant articles, at least 50% of these items were missed. Of these 11, eight of the journals' missing relevant articles were in publication category A, i.e. a case report, clinical trial, comparative study, evaluation study, journal article, multicenter study, research support or review.

**Table 3:** Breakdown of relevant records missed when a key search term was omitted.

	Relevant records	Relevant records missed	Missed records without an abstract	Missed records publication category A
1. Canadian Medical Association Journal	4	100% (4)	100% (4)	50% (2)
2. BMJ	25	92% (23)	96% (22)	13% (3)
3. American Journal of Public Health	6	83% (5)	0% (0)	100% (5)
4. The Lancet	9	78% (7)	100% (7)	29% (2)
5. Vaccine	4	75% (3)	0% (0)	100% (3)
6. AIDS Alert	6	67% (4)	100% (4)	0% (0)
7. Nature	6	67% (4)	100% (4)	0% (0)
8. Science	14	64% (9)	100% (9)	0% (0)
9. Influenza and Other Respiratory Viruses	10	60% (6)	17% (1)	83% (5)
10. Eurosurveillance	29	59% (17)	12% (2)	88% (15)
11. The New England Journal of Medicine	10	50% (5)	100% (5)	80% (4)
12. Morbidity and Mortality Weekly Report	11	36% (4)	0% (0)	100% (4)
13. Weekly Epidemiological Record	10	30% (3)	100% (3)	100% (3)
14. The Lancet Infectious Diseases	5	20% (1)	100% (1)	0% (0)
15. Journal of Clinical Virology	9	11% (1)	0% (0)	100% (1)
16. Journal of Clinical Microbiology	4	0% (0)	0% (0)	0% (0)

The table covers the 16 journals in which at least three records were identified as relevant during the 10 weeks covered by the present study (Step 11).

Out of the total number of relevant records that would be missed in the 10 simulated searches, 75% were category A publications when we omitted “h1n1,” 23% for “swine,” 88% for “influenza” and “29%” for “flu” (data not shown in tables); 59% of the missed relevant records did not include an abstract. This was the case for only 29% of the relevant records that were never missed when a term was omitted.

## Discussion

This study set out to show the impact that the inconsistent naming of a disease, such as influenza A(H1N1), has for health experts when searching for scientific articles in PubMed and MEDLINE. In turn, this can affect researchers' ability to communicate up-to-date findings to decision-makers and ultimately shape public health interventions. It demonstrates that an average of between 13.6% and 20.6% of the relevant articles are not captured if just one keyword is left out of the search string. As a result, it is unlikely that all important research will reach the researchers, practitioners, and decision-makers who can utilise it.

Our focus in this study was on scientific articles. It is well known that translating the findings of such studies into actionable messages for decision-makers is a complicated task [8]. The next step, translating such messages into widely accepted evidence-based public health, has been described as one of the greatest challenges facing health promotion and disease prevention [9], as well as a “slow and often haphazard process” [10]. This process includes disseminating the findings. More than 15 years ago, Jonathan Lomas wrote that the dissemination of medical research requires collaboration between academics and medical organisations [11], and that it is not enough to just publish research findings. The flow of information must be targeted, tailored, and more aggressive than mere “diffusion.” These observations are certainly applicable to the 2009 influenza A(H1N1) pandemic, a situation in which the media, fuelled by rapid diffusion of information on the Internet, have set the agenda more often than not. And as we demonstrate, experts and decision-makers face an uphill battle in finding the most recent evidence due to inconsistent naming of an emerging disease compounded by the NLM's lag time in assigning MeSH. That said, the leading public health institutions involved in synthesizing real-time pandemic data may not directly depend on data published in scientific journals to inform public health messaging or to decide on immediate public health measures. However, for academic and public health institutions at all levels to react effectively and base their short and long-term decisions on the best available knowledge, relevant research and communications published in peer-reviewed journals need to reach these institutions as quickly as possible.

Developing a search strategy that will identify all relevant articles published in scientific journals is impractical [12] as all searchers have their own approaches to the process, based on their knowledge of the topic and their experience with the databases and other sources they use. For example, several leading medical journals have sought to facilitate easy access to research on the pandemic. *The Lancet* (http://www.thelancet.com/H1N1-flu), *The New England Journal of Medicine* (http://h1n1.nejm.org), *BMJ* (http://pandemicflu.bmj.com) and *Public Library of Science* (http://www.ploscurrents.org/influenza) have all developed online resource centres to help users find scientific information about the pandemic, including publications. The scientific community should warmly welcome such initiatives. However, these information retrieval resources have limited journal coverage compared to PubMed/MEDLINE.

The NLM has also developed a search string that is slightly different from the one used in this study, but with the same purpose: to identify records on the pandemic recently added to PubMed. It is featured on the PubMed homepage (http://www.pubmed.gov). As of 21 January 2010, this search string was:

(swine OR h1n1) AND (flu OR influenza OR virus OR outbreak OR pandemic) [13]

Comparing its results with those generated by our core search string (Step 1) reveals that the NLM string misses several relevant records (see Table 4).

**Table 4:** Relevant articles missed by the National Library of Medicine search string.

Title of record (journal title)	Publication types
StatFlu – a static modelling tool for pandemic influenza hospital load for decision makers (Eurosurveillance) [23]	Journal articleResearch support
Population-based simulations of influenza pandemics: validity and significance for public health policy (Bulletin of the World Health Organization) [24]	Journal articleResearch support
Ten things your emergency department should consider to prepare for pandemic influenza (Emergency Medicine Journal) [25]	Journal article
The limitations of point of care testing for pandemic influenza: what clinicians and public health professionals need to know (Canadian Journal of Public Health) [26]	Comparative studyEvaluation studyJournal article
Considerations for assessing the severity of an influenza pandemic (Weekly Epidemiological Record) [27]	Journal article

### Limitations

The principal limitation of this study is the difficulty of validating the results. It can be done in part by “hand-searching” all available journals during the 10-week study period. Yet that would involve individually searching not only the journals that returned results for our core search string, but *all* the journals in PubMed. As such it is clearly not an option. Moreover, since MEDLINE indexing can take several months, many journal issues from the study period are still not indexed in the database and would thus require physical inspection.

### Solutions

There are several ways to improve the success of PubMed searches for pandemic information. First, the NLM could provide journal publishers with the possibility of including a special alert tag when they upload a new citation to the PubMed database. This tag would indicate that the cited item includes information about a rapidly evolving situation such as a pandemic. If these tags were used consistently, they would greatly facilitate such searches. The tags could then be revised or removed when MeSH are added. To our knowledge, this approach has not previously been tested in the context of health related bibliographic databases. However, a similar concept is widely used for information sharing on Internet services like CiteULike (http://www.citeulike.org) and Delicious (http://www.del.icio.us), where users can add tags of their own choice to their papers and links.

Second, publishers should be more diligent about including short abstracts of the records they add to PubMed. As shown, a substantial portion of the relevant records that were missed did not have an abstract. An abstract increases the likelihood that a searcher will find – and utilise – relevant materials.

Third, as part of its pandemic preparedness planning, WHO should prioritize the prompt naming of new disease strains involved in outbreaks, after appropriate consultation with scientific experts, research librarians, communication experts, and perhaps linguists. Such action should not hinder the introduction of a new name at a later stage, as long as consistency in use is maintained. A recent study has shown that WHO was the most cited institution during the first days of the influenza A(H1N1) pandemic [14]. As such it plays a key role in determining and clarifying pandemic nomenclature. It is no small responsibility, given that the inability of (re)searchers to find relevant articles impedes the transfer of knowledge to experts and health policy-makers, to the potential detriment of public health.

Fourth, our own experience in creating and maintaining the influenza A(H1N1) web site for the WHO Regional Office for Europe (http://www.euro.who.int/influenza/ah1n1) underscores the importance of carefully and consistently translating key pandemic terms into languages other than English. Although not relevant for the PubMed database, this issue is important for searching the WHO site and other web sites in foreign languages.

Finally, to avoid delays in becoming acquainted with new research, we suggest that people who rely on access to the latest published research subscribe to the really simple syndication (RSS) feeds provided by most publishers and PubMed itself. This technology can be utilised in several ways to enhance the timeliness and retrieval of research updates (see Box S2 for an example).

### Conclusion

Researchers and other experts should realize that when they search for newly published scientific material at the beginning of a pandemic, such as the 2009 influenza A(H1N1) pandemic, it is highly probable that they will not retrieve all the relevant articles. Our study demonstrated that a search string that does not include a combination of terms that covers several of the names used for the pandemic will miss relevant publications indexed in PubMed/MEDLINE. Leaving out just one term from a search can result in missing as much as a third of the relevant articles at a given point in time. These findings can have significant implications for the communication and utilisation of pandemic information.

There are two main ways to remedy this deficiency. The first is to agree on a name earlier and clearly communicate it (and any subsequent changes) to the scientific community and media. The World Health Organization is perhaps best placed to lead this effort. The second solution is for the National Library of Medicine to implement faster indexing of publications that relate to a rapidly unfolding health crisis, as well as to provide publishers the possibility of adding alert tags for such articles.

## Supporting Information

Box S1 Search tags used for searching PubMed
(0.03 MB DOC)

Box S2 The WHO Regional Office for Europe RSS feed on influenza A(H1N1)
(0.03 MB DOC)
