# Supplementary material for: Searching PubMed during a Pandemic
Source: PLoS One. 2010 Apr 7;5(4):e10039. doi: 10.1371/journal.pone.0010039 (PMC2850925; doi:10.1371/journal.pone.0010039)
Supplement: Box S1 — Search tags used for searching PubMed (0.03 MB DOC) [file pone.0010039.s001.doc]

| **Box S1. Search tags used for searching PubMed**   | [all] | Includes all search fields (e.g. title, abstract and MeSH) except for place of publication and transliterated title. This is the default when no tag is specified. | | --- | --- | | [edat] | Entrez date. Date the record was added to the PubMed database. Date ranges are searched by inserting a colon (:) between each date, e.g. 2009/04/27:2009/05/03[edat]. | | [mh] | Medical subject heading (MeSH). MeSH terms describe the subject of every record in MEDLINE. | | [mhda] | MeSH date. The date the record was assigned MeSH and elevated from PubMed to MEDLINE. Until MeSH are assigned, the date is set to the Entrez date. Date ranges are searched in the same way as the Entrez date. | | [pmid] | PubMed unique identifier. Unique number assigned to each record when it is first entered into PubMed. | | [tiab] | Title and/or abstract. Words included in the title and, if available, the abstract. | |
| --- | --- | --- | --- | --- | --- | --- | --- | --- | --- | --- | --- | --- |
