# Supplementary material for: Searching PubMed during a Pandemic
Source: PLoS One. 2010 Apr 7;5(4):e10039. doi: 10.1371/journal.pone.0010039 (PMC2850925; doi:10.1371/journal.pone.0010039)
Supplement: Box S2 — The WHO Regional Office for Europe RSS feed on influenza A(H1N1) (0.03 MB DOC) [file pone.0010039.s002.doc]

| **Box S2. The WHO Regional Office for Europe RSS feed on influenza A(H1N1)**  On its influenza A(H1N1) pandemic web site, the WHO Regional Office for Europe has implemented an automatically generated list of updated records (http://www.who.euro.who/influenza/ah1n1/rssreader). This list is based on an RSS feed of the search results from our core search. Making this list available on the Internet makes it possible for the public, including researchers and policy-makers, to stay abreast of new articles on the pandemic as they are published.  We suggest further minimizing delays in finding new research by:   - identifying the journals that have published potentially relevant articles on the influenza A(H1N1) pandemic; - locating the RSS feeds of these journals and other major journals in the field of communicable diseases and “mixing” them into a single feed using a web-based aggregator or mashup service, e.g. Yahoo! Pipes (http://pipes.yahoo.com); and - filtering this composite feed so it only shows articles containing certain terms, e.g. “h1n1,” “swine,” “pandemic,” “epidemic,” or “outbreak.” The last four only in conjunction with “influenza” or “flu.”   Such feeds make it possible to stay informed about new articles before they are added to PubMed. One good example of an RSS feed on influenza A(H1N1) can be found at http://www.google.com/reader/shared/user/12553290924569535832/label/Influenza_A(H1N1)pdm. |
| --- |
